# ChemTS: an efficient python library for *de novo* molecular generation

**DOI:** 10.1080/14686996.2017.1401424

**Published:** 2017-11-24

**Authors:** Xiufeng Yang, Jinzhe Zhang, Kazuki Yoshizoe, Kei Terayama, Koji Tsuda

**Affiliations:** ^a^ Graduate School of Frontier Sciences, The University of Tokyo, Kashiwa, Japan.; ^b^ Department of Biosciences, INSA Lyon, Villeurbanne Cedex, France.; ^c^ RIKEN, Center for Advanced Intelligence Project, Tokyo, Japan.; ^d^ National Institute for Materials Science, Tsukuba, Japan.

**Keywords:** Molecular design, Monte Carlo tree search, recurrent neural network, python library, 60 New topics/Others, 404 Materials informatics / Genomics

## Abstract

Automatic design of organic materials requires black-box optimization in a vast chemical space. In conventional molecular design algorithms, a molecule is built as a combination of predetermined fragments. Recently, deep neural network models such as variational autoencoders and recurrent neural networks (RNNs) are shown to be effective in *de novo* design of molecules without any predetermined fragments. This paper presents a novel Python library ChemTS that explores the chemical space by combining Monte Carlo tree search and an RNN. In a benchmarking problem of optimizing the octanol-water partition coefficient and synthesizability, our algorithm showed superior efficiency in finding high-scoring molecules. ChemTS is available at https://github.com/tsudalab/ChemTS.

## Graphical Abstract

**Figure:**
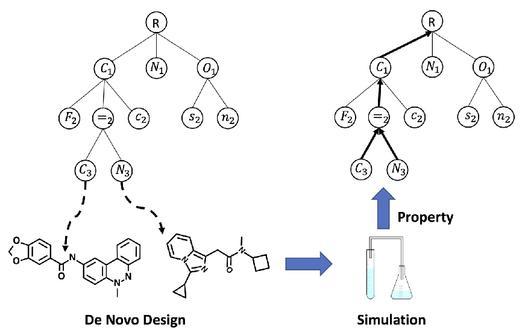

## 1. Introduction

In modern society, a variety of organic molecules are used as important materials such as solar cells [1], organic light-emitting diodes [2], conductors [3], sensors [4] and ferroelectrics [5]. At the highest level of abstraction, design of organic molecules is formulated as a combinatorial optimization problem to find the best solutions in a vast chemical space. Most computer-aided methods for molecular design build a molecule by a combination of predefined fragments (e.g. [6]). Recently, Ikebata et al. [7] succeeded *de novo* molecular design using an engineered language model of SMILES representation of molecules [8]. It is increasingly evident, however, that engineered models often perform worse than neural networks in text and image generation [9,10]. Gomez-Bombarelli et al. [11] were the first to employ a neural network called variational autoencoder (VAE) to generate molecules. Later Kusner et al. enhanced it to grammar variational autoencoder (GVAE) [12]. SMILES strings created by VAEs are mostly invalid (i.e. they do not translate to chemical structures); so, generation steps have to be repeated many times to obtain a molecule. Segler et al. [13] showed that a recurrent neural network (RNN) using long short-term memory (LSTM) [14] achieves a high probability of valid SMILES generation. In their algorithm, a large number of candidates are generated randomly and a black-box optimization algorithm is employed to choose high-scoring molecules. It is required to generate a very large number of candidates to ensure that desirable molecules are included in the candidate set. Optimization in a too large candidate space can be inhibitively slow.

In this paper, we present a novel Python library ChemTS to offer material scientists a versatile tool of *de novo* molecular design. The space of SMILES strings is represented as a search tree where the *i*th level corresponds to the *i*th symbol. A path from the root toa terminal node corresponds to a complete SMILES string. Initially, only the root node exists and the search tree is gradually generated by Monte Carlo tree search (MCTS) [15]. MCTS is a randomized best-first search method that showed exceptional performance in computer Go [16]. Recently, it has been successfully applied to alloy design [17]. MCTS constructs only a shallow tree and downstream paths are generated by a rollout procedure. In ChemTS, an RNN trained by a large database of SMILES strings is used as the rollout procedure. In a benchmarking experiment, ChemTS showed better efficiency in comparison to VAEs, creating about 40 molecules per minute. As a result, high-scoring molecules were generated within several hours.

## 2. Method

ChemTS requires a database of SMILES strings and a reward function *r*(*S*) where $S=\{s_{1},\ldots ,s_{T}\}$ is an input SMILES string. Our definition of SMILES strings contains the following symbols representing atoms, bonds, ring numbers and branches: $s_{t}\in$ {C, c, o, O, N, F, [C@@H], n, -, S,Cl, [O-],[C@H], [NH+],[C@], s, Br, [nH], [NH3+], [NH2+], [C@@], [N+], [nH+], [S@], [N-], [n+],[S@@], [S-], I, [n-], P, [OH+],[NH-], [P@@H], [P@@], [PH2], [P@], [P+], [S+],[o+], [CH2-], [CH-], [SH+], [O+], [s+], [PH+], [PH], [S@@+], /,=, #, 1,2,3,4,5,6,7,8,9,(, ),}. In addition, we have a terminal symbol $. The reward function involves first principle or semi-empirical calculations and describes the quality of the molecule described by *S*. If *S* does not correspond to a valid molecule, *r*(*S*) is set to an exceptionally small value. We employ *rdkit* (www.rdkit.org) to check if *S* is valid or not. Before starting the search, an RNN is trained by the database and we obtain the conditional probability $P(s_{t}|s_{1},\ldots ,s_{t-1})$ as a result. The architecture of our RNN is similar to that in [13] and will be detailed in Section 2.1.

**Figure 1.**
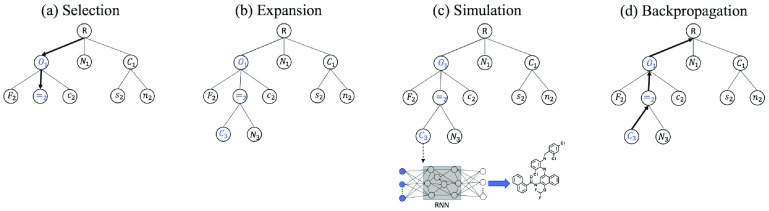
Monte Carlo tree search. (a) Selection step: the search tree is traversed from the root to a leaf by choosing the child with the largest UCB score. (b) Expansion step: 30 children nodes are created by sampling from RNN. (c) Simulation step: paths to terminal nodes are created by the rollout procedure using RNN. Rewards of the corresponding molecules are computed. (d) Backpropagation step: the internal parameters of upstream nodes are updated.

MCTS creates a search tree, where each node corresponds to one symbol. Nodes with the terminal symbol are called terminal nodes. Starting with the root node, the search tree grows gradually by repeating the four steps, selection, expansion, simulation and backpropagation (Figure 1). Each intermediate node has an upper confidence bound (UCB) score that evaluates the merit of the node [15]. The distinct feature of MCTS is the use of *rollout* in the simulation step. Whenever a new node is added, paths from the node to terminal nodes are built by a random process. In computer games, it is known that uniformly random rollout does not perform well, and designing a better rollout procedure based on available knowledge is essential in achieving high performance [15]. Our idea is to employ a trained RNN for rollout. A node at level t-1 has a partial SMILES string $s_{1},\ldots ,s_{t-1}$ corresponding to the path from the root to the node. Given the partial string, RNN allows us to compute the distribution of the next letter $s_{t}$. Sampling from the distribution, the string is elongated by one. Elongation by RNN is repeated until the terminal symbol occurs. After elongation is done, the reward of the generated string is computed. In the backpropagation step, the reward is propagated backwards and the UCB scores of traversed nodes are updated. See [17] for details about MCTS.

**Figure 2.**
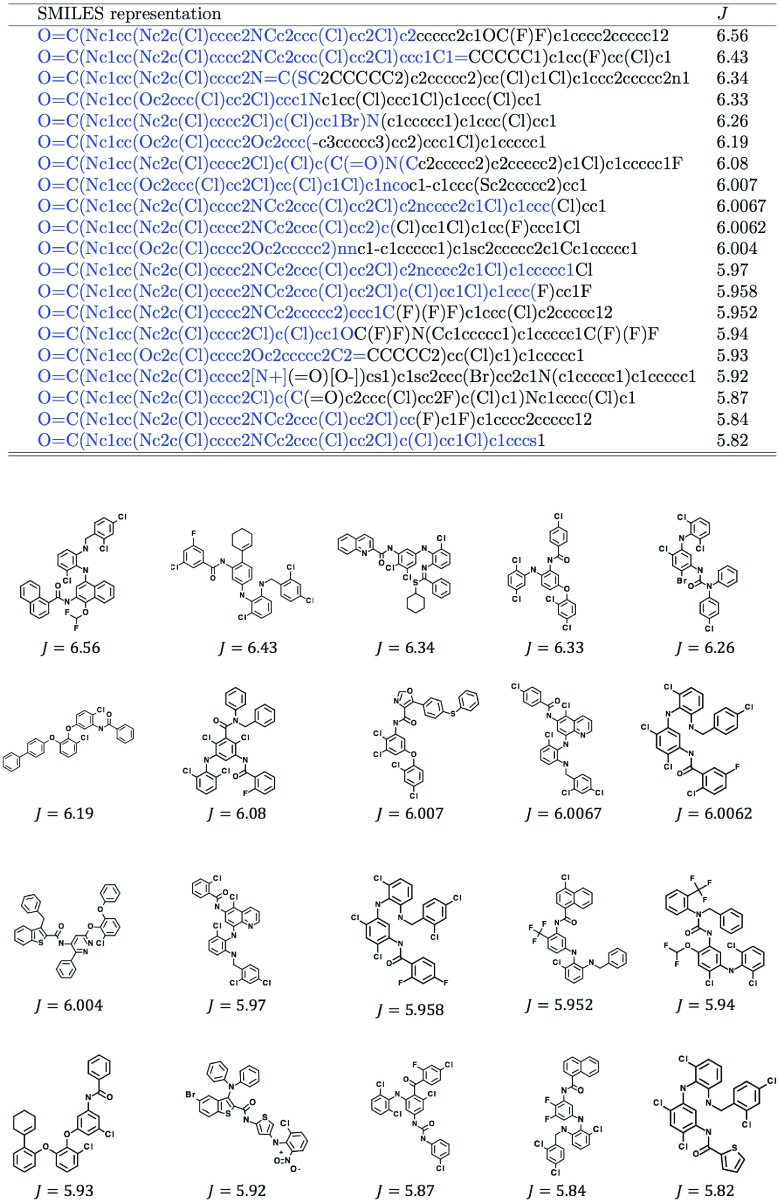
Best 20 molecules by ChemTS. Blue parts in SMILES strings indicate prefixes made in the search tree. The remaining parts are made by the rollout procedure.

**Table 1.** Maximum score *J* at time points 2,4,6 and 8 h achieved by different molecular generation methods.

Method	2 h	4 h	6 h	8 h	Molecules/Min
ChemTS	-pagination4.9±0.4	5.4±0.5	5.5±0.4	5.6±0.5	41±1.6
RNN+BO	-pagination3.5±0.3	4.5±0.2	4.5±0.2	4.5±0.2	8.3±0.0
Only RNN	-pagination4.5±0.3	4.6±0.3	4.8±0.3	4.8±0.3	41±1.4
CVAE+BO	-30±27	-1.4±2.2	-0.6±1.1	-0.0±0.9	0.1±0.1
GVAE+BO	-4.3±3.1	-1.3±1.7	-0.2±1.0	0.3±1.3	1.4±0.9

Notes: The rightmost column shows the number of generated molecules per minute. The average values and standard deviations over 10 trials are shown.

### 2.1. Recurrent neural network

Our RNN has a non-deterministic output: an input string $s_{1},\ldots ,s_{T}$ is mapped to probability distributions of output symbols $P\left(y_{1}\right),\ldots ,P\left(y_{T}\right)$. The RNN represents the function $h_{t}=f\left(h_{t-1},x_{t}\right)$, where $h_{t}\in R^{512}$ is a hidden state at position *t* and $x_{t}\in R^{64}$ is the one-hot coded vector of input symbol $s_{t}$. The function *f* is implemented by two stacked gated recurrent units (GRUs) [14], each with 256 dimensional hidden states. The input vector $x_{t}$ is fed to the lower GRU, and the hidden state of the lower GRU is fed to the upper GRU. The distribution of output symbol is computed as $P\left(y_{t}=j\right)=g_{j}\left(h_{t}\right)$, where $g_{j}$ is a softmax activation function depending only on the hidden state of the upper GRU.

Given *N* strings in the training set, we train the network such that it outputs a right-shifted version of the input. Denoted by $x_{\mathrm{it}}$, the one-hot coded vector of the *t*th symbol in the *i*th training string. The parameters in the network θ are trained to minimize the following loss function,

$$\min_{\theta }\sum_{i=1}^{N}\sum_{t=1}^{T-1}D\left(x_{it+1},P\left(y_{t}\right)\right),$$

where *D* denotes the relative entropy. Our RNN was implemented using Keras library (github.com/fchollet/keras), and trained with ADAM [18] using a batch size of 256. After the training is finished, one can compute $P(y_{t})$ from $s_{1},\ldots ,s_{t-1}$. It allows us to perform rollout by sampling the next symbol repeatedly.

## 3. Experiments

Following [11], we generate molecules that jointly optimize the octanol-water partition coefficient logP and the other two properties: synthetic accessibility [19] and ring penalty that penalizes unrealistically large rings. The score of molecule *S* is described as

(1) $$\begin{array}{c} J(S)=logP(S)-SA(S)-RingPenalty(S). \end{array}$$

The reward function of ChemTS is defined as

(2) $$\begin{array}{c} r\left(S\right)=\left\{\begin{array}{cc} \frac{J(S)}{1+|J(S)|} & \mathrm{Valid}\;\mathrm{SMILES}\\ -1.0 & \mathrm{otherwise}. \end{array}\right. \end{array}$$

ChemTS was compared with two existing methods CVAE [11] and GVAE [12] based on variational autoencoders. Their implementation is available at https://github.com/mkusner/grammarVAE. Both methods perform molecular generation by Bayesian optimization (BO) in a latent space of VAE. RNN, CVAE and GVAE were trained with approximately 250,000 molecules in ZINC database [20]. All methods were trained for 100 epochs. Training took 3.8, 9.4 and 33.5 h, respectively, on a CentOS 6.7 server with a GeForce GTX Titan X GPU. To evaluate the efficiency of MCTS, we prepared two alternative methods using RNN. One is simple random sampling using RNN, where the first symbol is made randomly and it is elongated until the terminal symbol occurs. The other is the combination of RNN and Bayesian optimization [21], where 4000 molecules are made a priori and Bayesian optimization is applied to find the best scoring molecule.

As shown in Table 1, effectiveness of each method is quantified by the maximum score *J* among all generated molecules at 2, 4, 6 and 8 h and the speed of molecules generation (i.e. the number of generated molecules per minute). VAE methods performed substantially slower than RNN-based methods, which reflects the low probability of generating valid SMILES strings. ChemTS performed best in finding high-scoring molecules, while the speed of molecular generation (40.89 molecules per minute) was only slightly worse than random generation by RNN (41.33 molecules per minute). The combination of RNN and BO could not find high-scoring molecules. Preparing more candidate molecules may improve the best score, but it would further slow down the molecular generation. In general, it is difficult to design a correct reward function when there are multiple objectives. So, it is important to generate many good molecules in a given time frame to allow the user to browse and select favourite molecules afterwards. See Figure 2 for the best molecules generated by ChemTS.

## 4. Conclusion

In this paper, we presented a new Python package for molecular generation. It will be further extended to include more sophisticated tree search methods and neural networks. Use of additional packages for computational physics such as *pymatgen* [22] allows the users to easily implement their own reward function. We look forward to see ChemTS as a part of the open-source ecosystem for organic materials development.
